# Molecular beacon based real-time PCR *p1* gene genotyping, macrolide resistance mutation detection and clinical characteristics analysis of *Mycoplasma pneumoniae* infections in children

**DOI:** 10.1186/s12879-022-07715-6

**Published:** 2022-09-06

**Authors:** Lifeng Li, Jiayue Ma, Pengbo Guo, Xiaorui Song, Mingchao Li, Zengyuan Yu, Zhidan Yu, Ping Cheng, Huiqing Sun, Wancun Zhang

**Affiliations:** 1 Henan International Joint Laboratory of Children’s Infectious Diseases, Children’s Hospital Affiliated to Zhengzhou University, Henan Children’s Hospital, Zhengzhou Children’s Hospital, Zhengzhou, China; 2 Department of Neonatology, Children’s Hospital Affiliated to Zhengzhou University, Henan Children’s Hospital, Zhengzhou Children’s Hospital, Zhengzhou, China

**Keywords:** *Mycoplasma pneumoniae*, Genotyping, Molecular beacon, Macrolide resistance, Clinical characteristics

## Abstract

**Background:**

*Mycoplasma pneumoniae* can be divided into different subtypes on the basis of the sequence differences of adhesive protein P1, but the relationship between different subtypes, macrolide resistance and clinical manifestations are still unclear. In the present study, we established a molecular beacon based real-time polymerase chain reaction (real-time PCR) *p1* gene genotyping method, analyzed the macrolide resistance gene mutations and the relationship of clinical characteristics with the genotypes.

**Methods:**

A molecular beacon based real-time PCR *p1* gene genotyping method was established, the mutation sites of macrolide resistance genes were analyzed by PCR and sequenced, and the relationship of clinical characteristics with the genotypes was analyzed.

**Results:**

The detection limit was 1–100 copies/reaction. No cross-reactivity was observed in the two subtypes. In total, samples from 100 patients with positive *M. pneumoniae* detection results in 2019 and 2021 were genotyped using the beacon based real-time PCR method and P1-1 *M. pneumoniae* accounted for 69.0%. All the patients had the A2063G mutation in the macrolide resistance related 23S rRNA gene. Novel mutations were also found, which were C2622T, C2150A, C2202G and C2443A mutations. The relationship between *p1* gene genotyping and the clinical characteristics were not statistically related.

**Conclusion:**

A rapid and easy clinical application molecular beacon based real-time PCR genotyping method targeting the *p1* gene was established. A shift from type 1 to type 2 was found and 100.0% macrolide resistance was detected. Our study provided an efficient method for genotyping *M. pneumoniae*, valuable epidemiological monitoring information and clinical treatment guidance to control high macrolide resistance.

**Supplementary Information:**

The online version contains supplementary material available at 10.1186/s12879-022-07715-6.

## Background

*Mycoplasma pneumoniae* (*M. pneumoniae*, MP) is a common pathogen that can cause moderate upper respiratory tract infection, severe lower respiratory tract infection, and extrapulmonary clinical symptoms such as encephalitis, Stevens-Johnson syndrome, myocarditis and hemolytic anemia [1]. The most common infection of *M. pneumoniae* is community-acquired pneumonia (CAP). Statistically, 10–40% of pneumonic pathogens in school-aged children and adolescents consists of *M. pneumoniae*, and 4–8% consists of *M. pneumoniae* in adults, whereas this proportion increased to 20–70% during the epidemic period [2].

The adhesion of respiratory epithelial cells through the attachment organelle of *M. pneumoniae* is a key step for colonization and pathogenesis [4]. P1 is the major component of the adhesin protein complex at the surface of the organelle, which is essential for cytoadherence of *M. pneumoniae* [13]. According to the sequence differences of the *p1* gene, *M. pneumoniae* can be divided into two large subtypes, type 1 and type 2, but the clinical significance of different subtypes is controversial. Although in vitro analysis of immunogenicity of different subtypes showed differences [14], the earlier reports on *M. pneumoniae* P1 typing showed no correlation with susceptibility and severity of clinical symptoms [15, 16]. However, severe pneumonia and additional extrapulmonary clinical manifestations were reported for type 1 *M. pneumoniae* infection manifestations [17]. In another study, type 2 *M. pneumoniae* pneumonia patients were reported with more neurological and cardiovascular symptoms [18]. Simultaneously, the dynamic change in the proportion of two subtypes of P1 may also be related to the periodic outbreak and epidemic of *M. pneumoniae* [5]. Studies on *M. pneumoniae* typing and antibiotic susceptibility analysis showed that different *p1* gene types may be associated with macrolide resistance to a certain degree, and type 2 strains may be more susceptible to macrolides [19, 20]. Hence, it is critical to monitor the molecular epidemiological features of *M. pneumoniae* since the genotypes may be related to macrolide susceptibility, disease severity and the periodic outbreak and epidemic of the pathogen.

The main treatments for *M. pneumoniae* infection are antibiotics. Due to the lack of a cell wall, *M. pneumoniae* is naturally resistant to antibiotics acting on the cell wall, such as β-lactam drugs, glycopeptides and fosfomycin, and it is also resistant to polymixins, sulfonamides, trimethoprim, rifampicin and linezolid. Although aminoglycosides, chloramphenicol and gentamicin have activity against *M. pneumoniae*, they are not recommended for clinical use [2, 3]. Macrolides restrained bacterial growth by binding of the 23S rRNA to inhibit protein synthesis, hence macrolides, tetracycline and fluoroquinolone have better performance for the clinical treatment of *M. pneumoniae* infection. Due to the possible impact on children's development, tetracycline and fluoroquinolone are not recommended for children. Hence, macrolides, such as erythromycin and azithromycin, serve as the primary choice for the clinical treatment of *M. pneumoniae* pneumonia in children. However, macrolide-resistant *M. pneumoniae* is gradually increasing worldwide, especially in Asia, showing a high rate of drug resistance [4–11]. In China, the drug resistance rate of macrolides can be as high as 100%, whereas it is lower than 12% in North America, Europe and Australia, and declined from 90% in 2010–2011 to 11% in 2018–2019 in Japan, which may be explained by a decrease in macrolide use and a shift in the prevalent genotype of *M. pneumoniae* from macrolide-resistant type 1 to the susceptible type 2 [12]. Studies have found that the main macrolide resistance mechanism in *M. pneumoniae* is the mutation in the 23S rRNA V region, in which A2063G and A2064G mutations lead to high level resistance, and mutations at A2067G and C2617G are associated with lower resistance [8]. Thus, it is necessary to perform epidemiological monitoring of *M. pneumoniae* in different regions to monitor local epidemic characteristics.

Generally, the classification of *M. pneumoniae* is mainly based on the differences between two repeated regions RepMP4 and RepMP2/3 contained in the *p1* gene. Commonly used methods for *p1* genotyping include nested PCR, PCR product restriction fragment length polymorphism (PCR–RFLP), rapid cycle PCR and real-time PCR high-resolution melt (HRM) genotyping assay. [1, 4, 21, 22]. Nested PCR, rapid cycle PCR and PCR–RFLP have high accuracy advantage, but are time-consuming and labor intensive. Compared with traditional PCR, real-time PCR has the advantages of high sensitivity and shorter time consumption by amplifying a small target. The real-time PCR HRM genotyping assay requires amplification of the 1900 bp long region of the *p1* gene and consists of a HRM collection procedure, which may require longer time to obtain genotyping result [22]. Hence, we aimed to establish a molecular beacon based real-time PCR genotyping method targeting the *p1* gene, which can obtain genotype results rapidly and is easy for clinical application. Meanwhile, we investigated the prevalent genotypes in Henan, China using the method established and analyzed the clinical significance of genotyping by analyzing the relationship between genotypes, macrolide resistance and clinical symptoms.

In the present study, we developed a new genotyping method that uses molecular beacon based real-time PCR for *M. pneumoniae p1* gene genotyping. We examined the prevalent genotypes in Henan, China using the method established and analyzed the mutation sites of drug resistance genes by PCR and sequencing. The relationship of the clinical symptoms with the subtypes and macrolide resistance of *M. pneumoniae* was analyzed.

## Methods

### Clinical sample collection and nucleic acid extraction

Samples were collected from children who visited Henan Children’s Hospital for *M. pneumoniae* detection in 2019 and 2021. *M. pneumoniae* was detected by amplification of 16S rRNA using SAT-MP kit (Shanghai Rendu Biotechnology Co, Ltd). The samples with positive results were stored in − 80 °C for further use. This study was approved by the Ethics Committee of the Henan Children’s Hospital. Nucleic acid was extracted from sputum, pharyngeal swab and alveolar lavage fluid of the patients using Shengxiang Biological nucleic acid extraction or purification kit (Changsha, China) following the manufacture’s instructions.

### Plasmid construction and extraction

The representative strain of *p1* subtype 1 (P1-1) was *M. pneumoniae* M129, the coding gene was MPN141, and the GenBank accession number was NC_000912.1. The representative strain of *p1* subtype 2 (P1-2) was *M. pneumoniae* FH, the coding gene was MPNE_RS00820, and the GenBank accession number was NC_017504.1. The partial sequences of *p1* subtype 1 (*M. pneumoniae* M129) and *p1* subtype 2 (*M. pneumoniae* FH) genes were inserted into plasmid pUC57 using primer pairs P1-1 F/R and P1-2 F/R (Table 1) to construct the recombinant plasmids, which were transformed into *Escherichia coli* TOP10 to obtain the recombinant strains (Sangon, China). The plasmids were extracted using the plasmid extraction kit (DP103) of Tiangen (Beijing, China). The plasmids were used as the positive control and used for the sensitivity analysis of the following assay.

**Table 1:** Primers used in the present study

Primer name	Sequence (5’-3’)
P1-1 F	GCTCTAGAGGTTTGAGTGGGGCTGCAC
P1-1 R	GGGGTACCGAGCAAAACATCGCGCGCC
P1-2 F	ACGCGTCGACTGGTTTGAGTGGGGCTGCAC
P1-2 R	CGGGATCCATCCAAGTGATCAACGCGGTC
M129 F	CGAACCGAGAGTGGTCAAAAT
M129 R	CGAACTGGAAAGGGCAGTAC
MB1	FAM -CCCCCTAGCGAGGAGTCGGGTCAGTCCAGGGGG-BHQ1
MB2	HEX-CGCGAACAACTCCGGTGACCAAGGCACTTCGCG-BHQ1
MP FH F	AGACAGCACTAACCAAACAGGC
MP FH R	CCGAACTGGAAGGGGCAG
M129C	GGACTGACCCGACTCCTCGCT
FHC	GTGCCTTGGTCACCGGAGTTG
MP 23S Sen	GTCTCGGCTATAGACTCGGTG
MP 23S Ant	GCTACAACTGGAGCATAAGAG

### Primers and molecular beacon probes design

National Center for Biotechnology Information (NCBI) was used to check the *p1* gene sequences of two *p1* gene subtypes. The sequences of two *p1* gene subtypes were compared through Bioedit software [23], the RepMP4 differential sequences were selected for primer design. Upstream and downstream primers for P1-1 and P1-2 were designed by Primer Premier 5 [24] and NCBI Primer-BLAST. Beacon Designer [25] was used to design molecular beacon sequences based on upstream and downstream primer amplification regions and their differential sequences, and RNA Structure [26], OligoAnalyzer 3.1 [27] and mFold [28] were used to predict their structures.

### Molecular beacon based real-time PCR for P1 gene genotyping

The *p1* gene containing plasmids were prepared using the gradient dilution method. Positive quality control plasmids were detected by real-time PCR to analyze the detection feasibility and sensitivity. The 20 µL detection reaction was composed of 10 µL qPCR premix (Vazyme, China), M129 F/M129 R/MP FH F/MP FH R 0.4 µL (final concentration was 0.2 µM), MB1/MB2 0.5 µL (final concentration was 0.25 µM), template 1µL, and 5.4 µL nuclease free water. Real-time PCR was used to detect the fluorescence signal of specific reaction, and the reaction conditions were as follows: 95 °C, 5 min; 40 cycles at 95 °C, 10 s, 37 °C, 30 s, 60 °C, 30 s. FAM and HEX signals were collected using the CFX96 thermal cycler (Biorad, USA). Amplification results of the clinical *M. pneumoniae* infection samples were drawn together using Origin 2016 using the amplification data exported [29].

### Detection of mutations in domain V of *Mycoplasma pneumoniae* 23S rRNA

The primers (Table 1) used to amplify the domain V of *M. pneumoniae* 23S rRNA gene were designed using Primer Premier 5 [24] and synthesized by Sangon (Shanghai, China). PCR was performed to amplify the fragments of the gene using the *M. pneumoniae* positive samples as templates with the enzyme (Vazyme, Nanjing, China). The PCR products were sequenced in Sangon (Shanghai, China) and compared with the reference 23S rRNA gene using the Bioedit software [23].

### Statistical analysis

The statistical analyses were performed using IBM SPSS statistics 25.0 (IBM Corp., Armonk, NY, USA). Normality of data was detected by Kolmogorov–Smirnov test. The statistical significance was determined at the two-tailed 0.1 level. The two groups were compared using a two-sample independent t-test or the Mann–Whitney U test. Means ± standard deviations (SDs) and median values (interquartile ranges: Q25–Q75) were used to present normally distributed data and skewed distribution data, respectively. The Chi-squared test was used for categorical variables. P < 0.05 was regarded as statistically significant.

## Results

### Molecular beacon based real time-PCR for *p1* gene genotyping assay

**Fig. 1:**
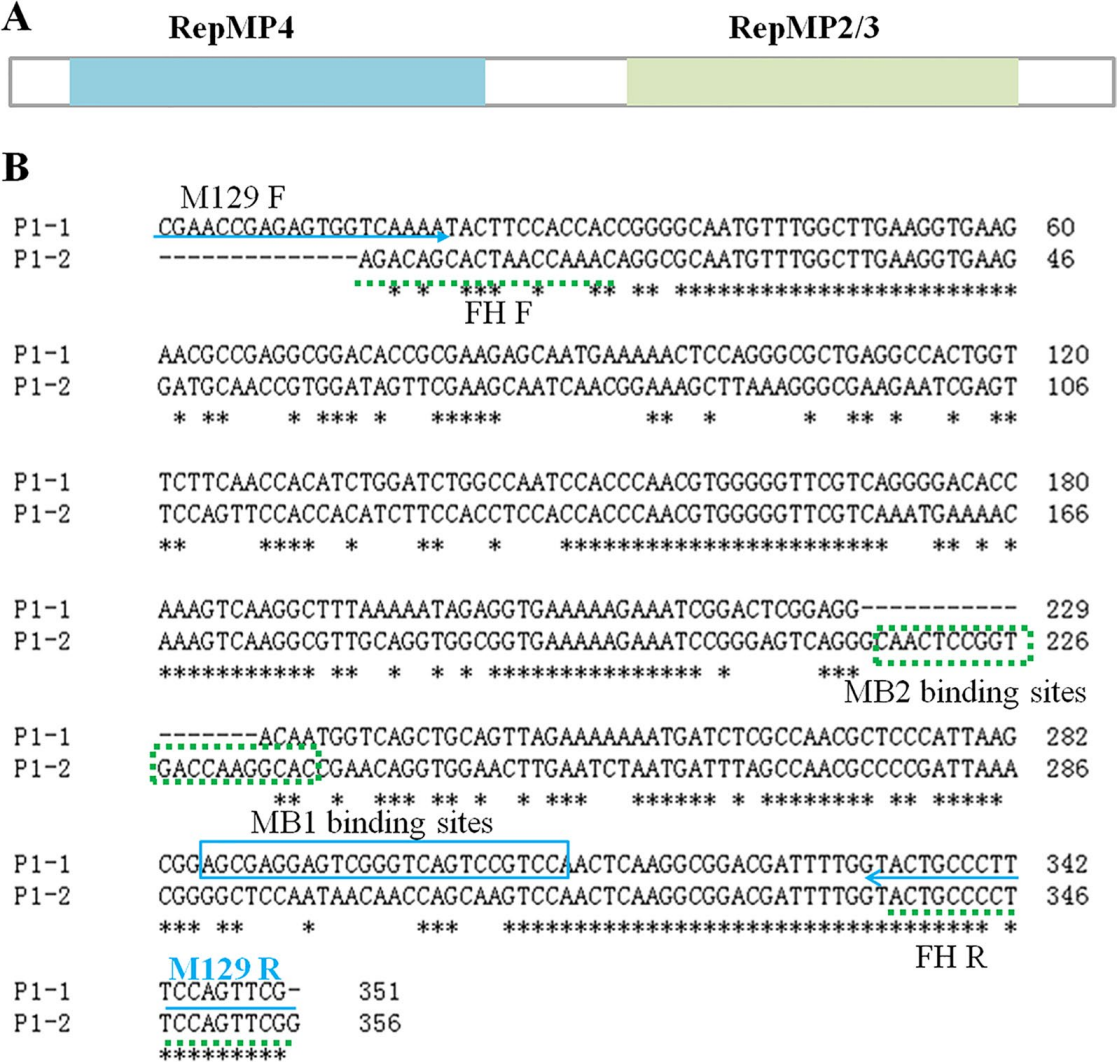
Composition of the* p1* gene and sequence alignments of primer binding sites. **A** The illustration of the *p1* gene indicating the RepMP4 and RepMP2/3 regions; **B** Sequence alignments of type 1 and 2 primer and molecular beacon binding sites. Sequence alignment picture was drawn using the tool reported [38]. Blue solid lines were used to indicate binding sequences of type 1 primer and molecular beacon. Green dotted lines were used to indicate binding sequences of type 2 primer and molecular beacon

To establish the beacon based genotyping method, the primer sets M129 F/M129 R and MP FH F/MP FH R were designed to amplify the two *p1* gene RepMP4 regions, and MB1/MB2 were designed to have typical secondary structures with FAM/HEX fluorescence labels (Table 1, Fig. 1 and Additional file 1: Fig. S1). The melting temperature (*T*_m_) was the temperature at which half of the hairpin structure was unfolded into single-stranded DNA and the fluorescence signals reached half of the peak value. Tm values for two beacon probes were determined, which was 59 °C for MB1 and 58 °C for MB2 (Additional file 1: Fig. S1). To determine the optimal reaction temperature of the molecular beacons, the fluorescence signal changes of MB1 and MB2 in the presence and absence of the targets were detected by PCR by increasing temperature from 10 °C to 92 °C. The temperature corresponding to the maximum difference of fluorescence signal intensity in the presence and absence of the targets was the optimal reaction temperature, which was 30 °C for MB1 and 40 °C for MB2, hence 37 °C was used as the annealing temperature of the real-time PCR reaction.

**Fig. 2:**
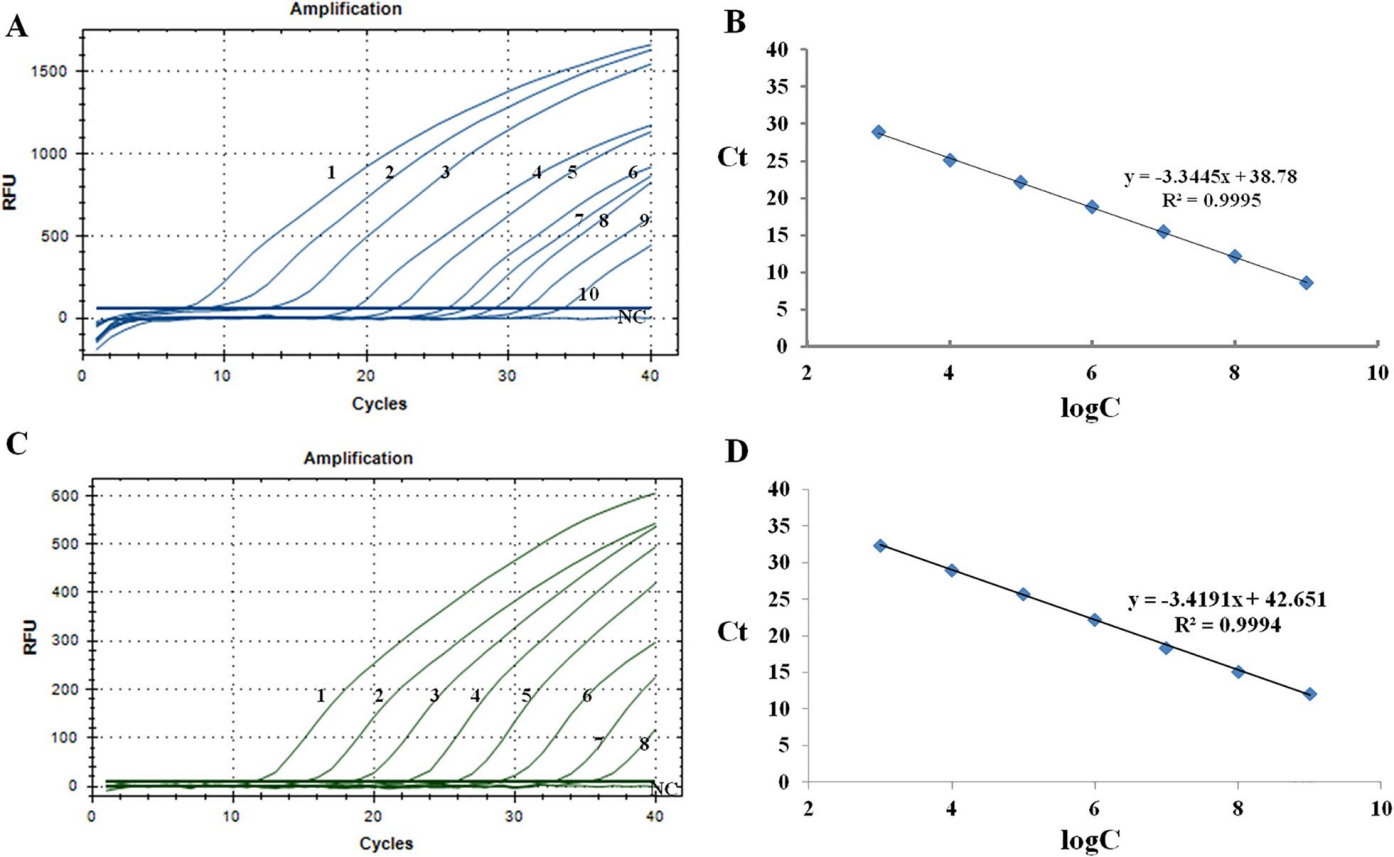
Amplification results of gradient positive quality control DNA. **A** The results of P1-1 amplification; **B** The standard curve of P1-1 amplification; **C** The results of P1-2 amplification; **D** The standard curve of P1- 2 amplification. The numbers 1–10 represent 1 × 10^9^copies/μL, 1 × 10^8^ copies/μL, 1 × 10^7^copies/μL, 1 × 10^6^ copies/μL, 1 × 10^5^copies/μL, 1 × 10^4^ copies/μL, 1 × 10^3^copies/μL, 1 × 10^2^ copies/μL, 1 × 10^1^ copies/μL and 1 copies/μL, respectively. NC was the negative control

Positive control plasmids carrying the *p1* gene with a gradient concentration were used to assess the sensitivity of the beacon based real-time PCR method (Fig. 2). The detection limit was 1 × 10^0^ copies for P1-1, and when the copy number of standard DNA ranged from 1 × 10^3^ copies/μL to 1 × 10^9^ copies/μL, there was a good linear relationship. The detection limit was 1 × 10^2^ copies for P1-2, and when the copy number of standard DNA ranged from 1 × 10^3^ copies/μL to 1 × 10^9^ copies/μL, there was a good linear relationship. No amplification was found when the two pairs of primers and probes were cross-examined. Eight samples were genotyped using the method in the present study and the reported nest-PCR method [4], the two assays were in perfect agreement with five P1-1 genotypes and three P1-2 genotypes. This indicated that the method could be used to the following genotyping of *M. pneumoniae* infection.

### Genotyping of clinical *M. pneumoniae* infection samples

**Fig. 3:**
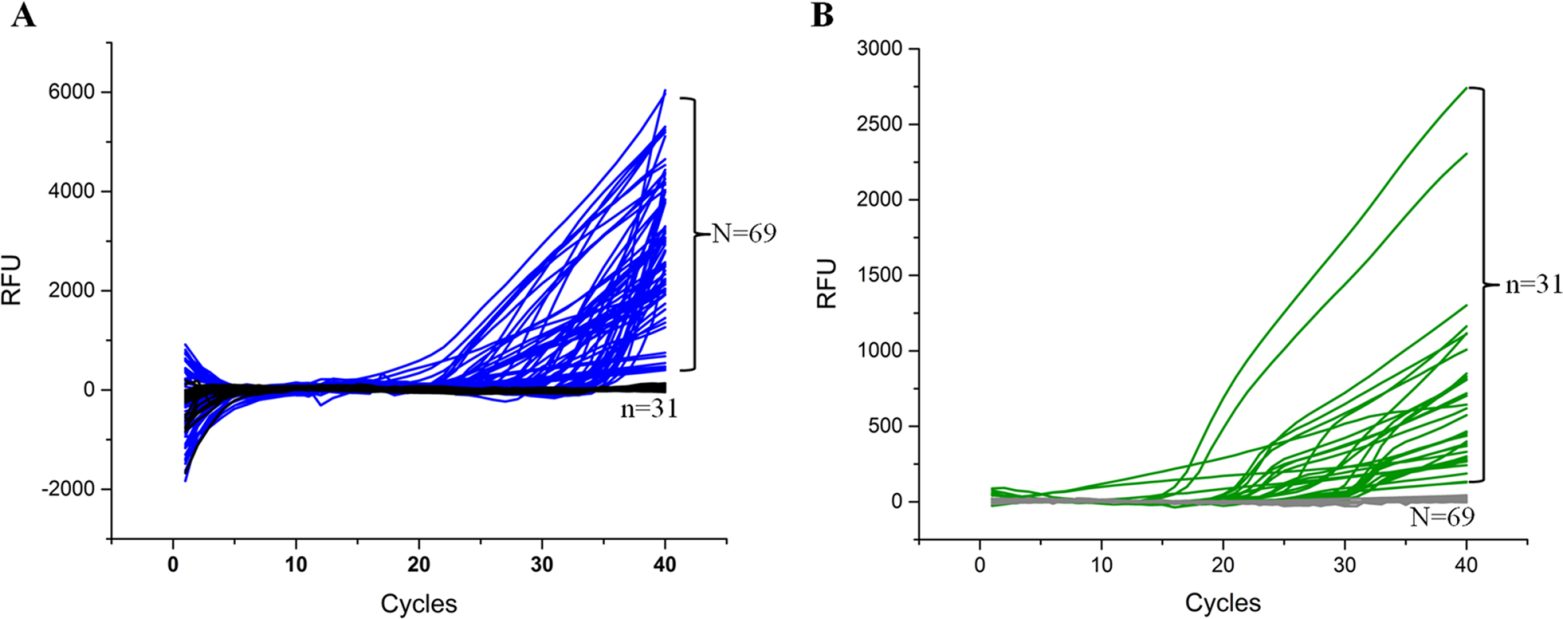
Amplification results of the 100 clinical *M. pneumoniae* infection samples. **A** The amplification results of FAM signals with 69 positive samples and 31 negative results; **B** The amplification results of HEX signals with 31 positive samples and 69 negative results. The figure was drawn using Origin 2016 using the results exported

In total, the samples were obtained from 100 patients with positive *M. pneumoniae* detection results in 2019 and 2021 including 59 boys and 41 girls (Table 2). There were 69 patients infected with P1-1 type *M. pneumoniae*, which included 40 boys and 29 girls; 31 patients with P1-2 M*. pneumoniae*, which included 19 boys and 12 girls. Moreover, the median age of patients were 4.90 years, 4.65 and 5.47 years in P1-1 and P1-2 groups respectively. The amplification results of 100 samples were shown in Fig. 3. To further verify the accuracy of our method, we verified the genotyping results by our method by analyzing half of the samples (50 of 100) using nest-PCR method, and the results were consistent with the method established in this study. No significant difference was observed in gender and age composition between the two groups. There was a significant difference between the *M. pneumoniae* infection group in 2019 and 2021 (P = 0.010) where the infection rate of P1-1 was higher than P1-2.

**Table 2:** Genotyping results of *M. pneumoniae* positive clinical samples

	P I-I	P I-II	Total	χ^2^	p
2019	55 (76.4%)	17 (23.4%)	72	6.653	0.010
2021	14 (50.0%)	14 (50.0%)	28		
Total	69 (69.0%)	31 (31.0%)	100

### Macrolide resistance mutation detection in the 23S rRNA gene

To analyze the macrolide resistance, the samples with genotyping results were further used to amplify the 23S rRNA gene and analyze the mutations in the domain V by sequencing. Among the 100 *M. pneumoniae* samples, all were found to have A2063G mutation compared with the sequence of the reference M129 strain. Two *M. pneumoniae* strains contained another C2622T mutation (2.0%). One *M. pneumoniae* strain had C2150A and C2202G mutation, which was subtype 2 genotype. One *M. pneumoniae* strain had C2443A mutation. The other three with new mutations belonged to the genotype P1-1.

### Clinical characteristics of pediatric *M. pneumoniae* infection

To analyze the relationship of *p1* gene genotyping and the clinical characteristics of *M. pneumoniae* infection, the demographics, clinical manifestations and laboratory examination results were analyzed and shown in Table 3. Aside from a higher lymphocyte count in the P1-1 group compared to the P1-2 group (2.83 [2.08–3.6] × 10^9^ cells/L vs 2.54 [1.31–2.99] × 10^9^ cells/L; Z = -2.132; P = 0.033), no significant difference was observed in the clinical symptoms analyzed between the two groups.

**Table 3:** Clinical characteristics of *M. pneumoniae* infection children

Variable	Total (n = 100)	P I-I (n = 69)	P I-II (n = 31)	t/Z/χ^2^	p
Male/Female	100	40/29	19/12	− 0.311	0.756
Age (years)	4.90	4.65 ± 2.16	5.47 ± 2.80	− 1.606	0.112^a^
Leukocyte count (× 10^9^ cells/L)	9.69	10.14 (7.23–10.80)	8.7 (5.94–10.14)	− 1.170	0.242
Neutrophil count (× 10^9^ cells/L)	6.18	6.53 (4.03–7.14)	5.38 (2.97–6.97)	− 0.332	0.740
Lymphocyte count (× 10^9^ cells/L)	2.74	2.83 (2.08–3.60)	2.54 (1.31–2.99)	− 2.132	0.033
CRP (mg/L)	25.11	25.43 (7.28–35.75)	24.45 (3.83–26.42)	− 1.108	0.268
PCT	0.242	0.200 (0.060–0.197)	0.229 (0.085–0.210)	− 0.565	0.572
Length of hospitalization (days)	11.40	11.35 (9–13)	11.52 (8–15)	− 0.193	0.847
Duration of fever (days)	6.79	7 (5.00–8.25)	6.29 (4.00–8.75)	− 0.429	0.668
Thermal spike (℃)	39.43	39.42 (39.00–39.90)	39.45 (39.00–40.00)	− 0.634	0.526
Pulmonary manifestations					
Positive (n, %)	52	35 (50.8%)	17 (54.8%)	0.145	0.703^b^
Negative (n, %)	48	34 (49.3%)	14 (45.2%)		
A: Pleural effusion	32	20	12	− 0.959	0.337
B: Pulmonary atelectasis	2	1	1	− 0.584	0.559
C: Pleuritis	1	1	0	− 0.670	0.503
D: Respiratory insufficiency; Respiratory failure	10	7	3	− 0.072	0.943
E: Pulmonary consolidation	27	17	10	− 0.790	0.430
F: Pneumothorax	3	2	1	− 0.088	0.930
G: Severe pneumonia	5	4	1	− 0.543	0.587
H: Pertussis syndrome	3	2	1	− 0.088	0.930
Extrapulmonary manifestations					
Positive (n, %)	49	33 (47.8%)	16 (51.6%)	0.123	0.726^b^
Negative (n, %)	51	36 (52.4%)	15 (48.4%)		
A: Digestive system					
A1: Peritoneal effusion	14	10	4	− 0.211	0.833
A2: Others*	19	13	6	− 0.060	0.952
B: Cardiovascular system	2	2	0	− 0.953	0.341
C: Nervous system	3	2	1	− 0.088	0.930
D: Blood-Lymphatic system					
D1: Sepsis	3	1	2	− 1.349	0.177
D2: Lymphadenectasis	20	12	8	− 0.968	0.333
D3: Anemia	2	2	0	− 0.953	0.341
E: Rash	4	3	1	− 0.263	0.792
F: Electrolyte disturbance	3	1	2	− 1.349	0.177
G: Immune system (allergy**,** immunodeficiency)	6	6	0	− 1.685	0.092
H: Urinary system	2	2	0	− 0.953	0.341
Two systems	4	3	1	− 0.263	0.792
Three systems	1	1	0	− 0.670	0.503

*Hepatomegaly, hepatic injury, abnormal liver function, Enteritis and gastrointestinal dysfunction

^a^Two-sample independent t-test

^b^Chi-squared test

## Discussion

In the present study, we described the development of a molecular beacon based *M. pneumoniae* genotyping method based on real-time PCR targeting *p1* gene, in which one reaction can detect two genotypes P1-1 and P1-2. First, the accuracy of this method was evaluated by comparing the results with the ones generated by nest-PCR [4]. Further, 100 *M. pneumoniae* infection samples were genotyped using this molecular beacon based method, the mutations in the domain V of 23S rRNA gene were analyzed by PCR and sequencing, and the clinical significances of genotyping were analyzed.

The reasons why *M. pneumoniae* infection can lead to different manifestations are not clear. Genotypes are associated with the clinical outcomes were reported [17, 18]. Hence, we conducted the genotyping of *M. pneumoniae* infections to further analyze its relationship with clinical symptoms in the present study. According to the sequence differences of repetitive elements RepMP2/3 and RepMP4 in the P1 protein gene, *M. pneumoniae* can be classified into subtype 1 and subtype 2 two major genotypes [30]. HRM analysis based real-time PCR was reported to distinguish two subtypes of P1 protein gene by amplifying the 1.9 kb fragment [22]. Compared to the HRM analysis based real-time PCR method, our method had a shorter amplification fragment and hence needed a shorter time ( about 1.5 h vs 2.5 h) to obtain the genotyping result. Additionally, our method can quantify the sample according to the standard curve. PCR–RFLP method was also used in the P1 gene genotyping, which could detect the subtypes by PCR amplification and agarose gel electrophoresis [4]. In this study, we developed a molecular beacon probes based real-time PCR method that can identify two subtypes by detection of different fluorescence signals in the amplification process. The genotyping results by our method were consistent with the data generated by the nest-PCR method. Hence, we used the method established to further analyze the genotype of *M. pneumoniae* infection samples in this study.

Different and changing ratios of the P1-1 and P1-2 subtype *M. pneumoniae* infections were reported worldwide [4, 22], and the incidence of P1-1 infections were usually more than P1-2 (Table 4). Genotyping is crucial for molecular epidemiological studies and the development of an effective vaccine [31]. In total, 69 (69.0%) of 100 children analyzed in this study were infected with P1-1 M*. pneumoniae* whereas the rate of P1-1 in 2019 was 76.4% and 50.0% in 2021. In 2015, 92.0% type 1 strain was reported on the basis of P1 gene PCR–RFLP analysis among 71 adults in Zhejiang province [32]. Zhao et al. performed a multicenter study analyzing molecular characteristics of *M. pneumoniae* by genotyping 154 isolates from 5 cities in mainland China in 2017–2018 and found that type 1 accounted for 76.6%, 23.4% for type 2 strains, and a large variance was found ranging from 100% type 1 in Jilin to 45.5% in Jinan [33]. Jiang et al. reported 57.1% type 1 *M. pneumoniae* infection by nested PCR from children with pneumonia in Qingdao, China, in 2019 [34]. Guo et al. analyzed the molecular features of *M. pneumoniae* isolates in paediatric inpatients in Weihai, China in 2019 and found that genotype 2 was identified in 42 isolates of 82 culture-positive samples [35]. Whistler et al. reported type 1 genotype *M. pneumoniae* accounted for a ratio of 61.8% (97/157) in the rural populations of Thailand from 2009 to 2012, and no macrolide resistance mutations were detected [36]. Kenri et al. found that the genotypes changed periodically in Japan where type 1 *M. pneumoniae* strains reduced from 100% of the strains isolated in 2012 to 8.3% in 2018 [37]. Hence, *p1* subtype 1 was the prevalent genotypes in several regions analyzed, and different epidemiological genotypes were distributed in different regions in China and other countries, whereas the prevalent genotypes of Japan indicated a substantial periodical change in the epidemiological features of *M. pneumoniae*.

**Table 4:** Distribution of *p1* gene subtypes of *M. pneumoniae* infection in different regions

Region	Year	Genotyping results	References
Henan, China	2019/2021	P1 subtype 1:76.4% in 2019 and 50.0% in 2021	This study
Zhejiang, China	2015	P1 subtype 1: 92.0%	[32]
Multicenter, China	2017–2018	Overall P1 subtype 1: 76.6%; P1 subtype 1: 100.0% in Jilin and 45.5% in Jinan	[33]
Qingdao, China	2019	P1 subtype 1: 57.1%	[34]
Weihai, China	2019	P1 subtype 2: 51.2%	[35]
Thailand	2009–2012	P1 subtype 1: 61.8%	[36]
Japan	2012–2018	P1 subtype 1: 100.0% in 2012 and 8.3% in 2018	[37]

As an important causative pathogen in CAP, *M. pneumoniae* increased in macrolide resistance. The high resistance rate was reported in East Asia, which was 81.6% in Japan, 87.2% in Korea, and 90% to 100% in China [10]. In our study, the macrolide resistance rate was 100.0% and all had the high resistance related A2063G mutation, which was consistent with a study in Qingdao, China in 2021 [34]. An earlier study in Zhejiang, China also reported A2063G mutation in all of the 71 *M. pneumoniae* strains isolated from adults with CAP in 2015 [32]. It is noteworthy that high resistance rate was also found in the type 2 strains in our study, while type 2 strains were related with the lower macrolide resistance rate in other studies reported [19, 20, 37]. Meanwhile, new mutations were found in the samples, which were C2622T, C2150A, C2202G and C2443A. Among four new mutations found, three was in P1-1 group and one was P1-2 group. The strains were needed to analyze the concrete impact of the new mutations on the macrolide resistance levels. Therefore, the high resistance rate requires special attention and macrolide antibiotic use in the clinical practice should be adjusted accordingly to reduce selection stress for the pathogen. Acute reduction in macrolide-resistant *M. pneumoniae* infections among Japanese children was reported, which may indicate the importance of changing antibiotic usage and the impact of *p1* genotype distribution [20].

Despite advances in genotyping methods to characterize different *M. pneumoniae* strains, the relationship between different genotypes and specific clinical outcomes it is still unclear. Hence, we analyzed the relationship between P1-1 genotypes and clinical characteristics. Although the majority clinical outcomes of infections caused by P1-1 and P1-2 subtype *M. pneumoniae* isolates are not significantly different, patients infected with P1-1 isolates had a higher lymphocyte count (2.83 × 10^9^ cells/L). Fan et al. analyzed 304 cases of type 1 *M. pneumoniae* and 30 cases of type 2 *M. pneumoniae* infection (type 1 91.0%) in children with pneumonia, and found that children infected with type 1 *M. pneumoniae* strain had a higher risk of developing severe pneumonia and with more extrapulmonary clinical manifestations [17]. Berlot et al. analyzed 356 cases of type 1 *M. pneumoniae* and 126 cases of type 2 *M. pneumoniae* pneumonia in children, which found that different types of *M. pneumoniae* infections in patients showed different clinical features. Type 2 *M. pneumoniae* pneumonia patients were with more neurological and cardiovascular symptoms, but patients infected with type 1 *M. pneumoniae* had other clinical manifestations, which indicated that different types of *M. pneumoniae* may have different pathogenicity [18].

Our study has limitations. First, the samples collected were geographically confined to Zhengzhou, Henan, China. Second, the number of analyzed *M. pneumoniae* strains were still relatively small, especially the samples in 2021, and it is not enough to analyze the impact of COVID-19 to epidemiological features of *M. pneumoniae*. Third, although the current design does not affect the results of this study, but variant 2d may be missed by our method, which should be noted in future uses.

## Conclusions

*M. pneumoniae* infection is common amongst pneumonia infections in the pediatric community with endemic prevalence worldwide. The prevalent genotypes vary over time and geographic location, which may or may not determine specific clinical outcomes. In summary, the molecular beacon based real-time PCR *p1* gene genotyping method was established, which had a detection limit of 1–100 copies depending on the subtypes. 100 *M. pneumoniae* infection samples from children with pneumonia in Zhengzhou were genotyped using this method. Type 1 *M. pneumoniae* was the main genotype. A shift from type 1 to type 2 was found and 100% macrolide resistance was detected. The macrolide resistance mutation A2063G could be detected in all the samples analyzed. Simultaneous, new mutations of C2622T, C2150A, C2202G and C2443A were found. Our study is an important addition to the molecular epidemiological features of *M. pneumoniae* in children in Henan, China combining the genotyping, macrolide resistance profile and analysis of clinical symptoms. Due to the high macrolide resistance rate found in our study and other reports in China, routine *M. pneumoniae* resistance profile detection would be critical for the control of *M. pneumoniae* macrolide resistance and guiding clinical treatments. In the future, national surveillance, long-term longitudinal and multi-center studies are needed to examine the molecular epidemiology information such as the periodic genotype shifts and the changes of antibiotic resistance to provide basis for the prevention and treatment of related diseases.

## Supplementary Information

**Additional file 1:** **Fig. S1**. Secondary structures and Tm values of two molecular beacons. **A** Secondary structures of MB1; **B** Secondary structures of MB2; **C** Tm values of MB1; **D** Tm values of MB2.

## Data Availability

The datasets used and/or analyzed during the current study are available from the corresponding author on reasonable request.

## Abbreviations

real-time PCR: Real-time polymerase chain reaction
MP: *Mycoplasma pneumoniae*, *M. pneumoniae*
CAP: Community-acquired pneumonia
PCR–RFLP: PCR product restriction fragment length polymorphism
HRM: High-resolution melt
NCBI: National Center for Biotechnology Information
SDs: Standard deviations
Tm value: Melting temperature values
