# Supplementary material for: Molecular beacon based real-time PCR p1 gene genotyping, macrolide resistance mutation detection and clinical characteristics analysis of Mycoplasma pneumoniae infections in children
Source: BMC Infect Dis. 2022 Sep 6;22:724. doi: 10.1186/s12879-022-07715-6 (PMC9447981; doi:10.1186/s12879-022-07715-6)

**Fig. S1 Secondary structures and Tm values of two molecular beacons.** (A) Secondary structures of MB1; (B) Secondary structures of MB2; (C) Tm values of MB1; (D) Tm values of MB2.


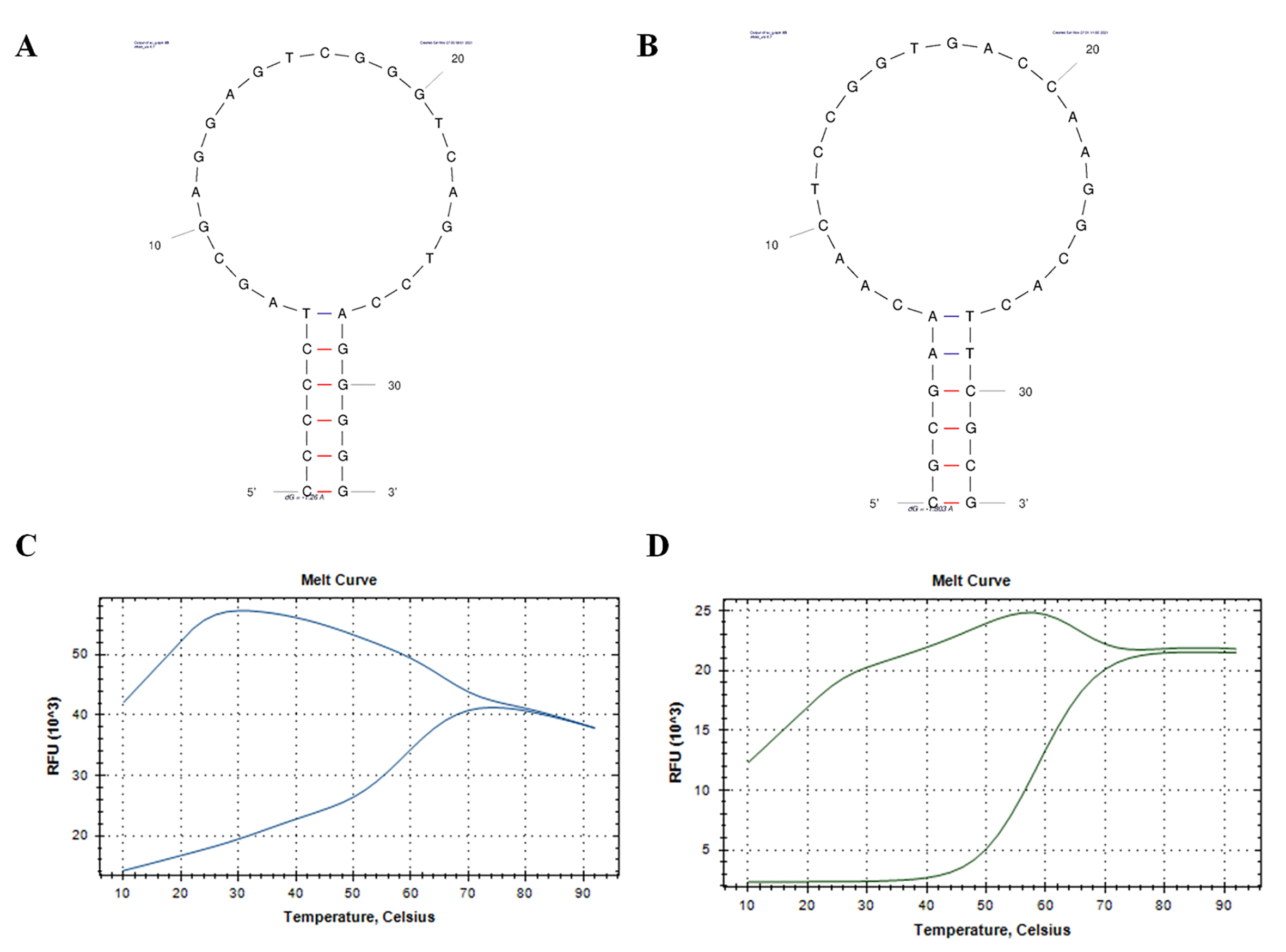

Supplement: Supplementary file 1 — Additional file 1: Fig. S1. Secondary structures and Tm values of two molecular beacons. A Secondary structures of MB1; B Secondary structures of MB2; C Tm values of MB1; D Tm values of MB2. [file 12879_2022_7715_MOESM1_ESM.docx]
